# STIM1 Is a Novel Component of ER-*Chlamydia trachomatis* Inclusion Membrane Contact Sites

**DOI:** 10.1371/journal.pone.0125671

**Published:** 2015-04-27

**Authors:** Hervé Agaisse, Isabelle Derré

**Affiliations:** Department of Microbial Pathogenesis, Yale School of Medicine, New Haven CT, United State of America; Penn State Hershey College of Medicine, UNITED STATES

## Abstract

Productive developmental cycle of the obligate intracellular bacterial pathogen *Chlamydia trachomatis* depends on the interaction of the replicative vacuole, named the inclusion, with cellular organelles. We have recently reported the formation of ER-Inclusion membrane contact sites (MCSs), where the endoplasmic reticulum (ER) is in apposition to the inclusion membrane. These platforms contain the *C*. *trachomatis* inclusion membrane protein IncD, the mammalian ceramide transfer protein CERT and the ER resident proteins VAPA/B and were proposed to play a role in the non-vesicular trafficking of lipids to the inclusion. Here, we identify STIM1 as a novel component of ER-Inclusion MCSs. STIM1, an ER calcium (Ca^2+^) sensor that relocate to ER-Plasma Membrane (PM) MCSs upon Ca^2+^ store depletion, associated with *C*. *trachomatis* inclusion. STIM1, but not the general ER markers Rtn3C and Sec61ß, was enriched at the inclusion membrane. Ultra-structural studies demonstrated that STIM1 localized to ER-Inclusion MCSs. Time-course experiments showed that STIM1, CERT and VAPB co-localized throughout the developmental cycle. By contrast, Orai1, the PM Ca^2+^ channel that interacts with STIM1 at ER-PM MCSs, did not associate with *C*. *trachomatis* inclusion. Upon ER Ca^2+^ store depletion, a pool of STIM1 relocated to ER-PM MCSs, while the existing ER-Inclusion MCSs remained enriched in STIM1. Finally, we have identified the CAD domain, which mediates STIM1-Orai1 interaction, as the minimal domain required for STIM1 enrichment at ER-Inclusion MCSs. Altogether this study identifies STIM1 as a novel component of ER-*C*. *trachomatis* inclusion MCSs. We discuss the potential role(s) of STIM1 during the infection process.

## Introduction

*Chlamydia trachomatis* is a Gram-negative obligate intracellular bacterial pathogen that infects ocular and genital epithelial surfaces. Upon entry into the host cells, the bacteria reside in a membrane bound compartment, named the inclusion. *C*. *trachomatis* developmental cycle lasts 2 days and begins with the entry of the infectious form of the bacteria (i.e. EB, Elementary Body) into the host cell. After differentiation into the replicative form (i.e. RB, Reticulate Body), *C*. *trachomatis* undergoes several rounds of bacterial replication, leading to the expansion of the inclusion. Midway through the cycle, RBs start to transition back into EBs. The exit of EBs from the host cell completes the cycle and allows for infection of the neighboring cells [1, 2].

During co-evolution of *C*. *trachomatis* with the mammalian host, the *C*. *trachomatis* genome has been reduced to less than 1,000 genes. The bacteria therefore rely on intercepting host cellular processes to complete its developmental cycle [3]. Independent studies have described the association of *C*. *trachomatis* inclusion with the endoplasmic reticulum (ER) [4–7]. Ultra-structural analysis revealed multiple patches of ER associated with the inclusion membrane. The ER and inclusion membranes are 10–20 nm apart, which is reminiscent of previously described membrane contact sites (MCSs) between the ER and various organelles or the plasma membrane (PM) [8–11]. We have therefore proposed that ER-Inclusion MCSs are formed during *C*. *trachomatis* infection [5]. The *C*. *trachomatis* inclusion membrane protein IncD, the soluble ceramide transfer proteins CERT and the integral ER membrane proteins VAPA and VAPB are specifically enriched at ER-*C*. *trachomatis* inclusion MCSs [5, 6]. Analysis of the interaction between IncD, CERT and VAPB revealed that IncD promotes CERT recruitment to the inclusion membrane through interaction with the PH (Pleckstrin Homology) domain of CERT. In addition, the FFAT (diphenylalanine [FF] in an Acidic Tract) motif of CERT mediates CERT interaction with VAPB located in the ER [5, 12]. Based on these results above, the requirement for host sphingolipids acquisition by *C*. *trachomatis* [13–17] and the association of the sphingomyelin synthase 2 (SMS2) with the inclusion membrane [6], it was proposed that ER-Inclusion MCSs might participate in the synthesis of sphingomyelin directly at the inclusion membrane to facilitate its acquisition by the bacteria. The actual function of ER-Inclusion MCSs remains however to be demonstrated.

MCSs have been described between the ER and nearly every membrane bound organelles (Golgi, endosomes/lysosomes, lipid droplets, mitochondria and PM). Tethering and functional components participate in positioning the membranes of the contacting organelles in close apposition in order to facilitate the non-vesicular trafficking and exchange of small molecules such as lipids or calcium (Ca^2+^) (reviewed by [18, 19]). Extensive studies of ER-PM MCSs have revealed their central role in store-operated calcium entry (SOCE), a process by which depletion of ER Ca^2+^ store induces the activation of Ca^2+^ release-activated Ca^2+^ (CRAC) channels leading to an influx of Ca^2+^ at the plasma membrane (reviewed by [20–23]). This process is important for various host cellular functions such as gene expression or regulation of cell proliferation. It involves two major players, Orai1 and STIM1. Orai1 is a PM protein with four predicted transmembrane domains and the amino- and carboxy- termini facing the cytosol. Its oligomerization constitutes the Ca^2+^ channel. STIM1 is a single-pass (aa 214–234) ER membrane protein containing several domains important for its function. The luminal N-terminal contains a signal peptide (aa 1–23), an EF hands domain (aa 63–98) and a sterile alpha motif (SAM) (aa 131–200). The cytosolic C-terminal contains two coil-coiled domains (aa 247–340 and 363–389), a Ser/Pro-rich region (aa 600–629) and a Lys-rich region (aa 672–685). In resting cells, STIM1 localizes to the bulk of the ER. Upon ER Ca^2+^ store depletion, the unbinding of Ca^2+^ from the EF-hand, triggers STIM1 oligomerization, *via* the SAM and coil-coiled domains, resulting in the redistribution of STIM1 into puncta in close apposition to the PM (i.e. ER-PM MCSs) where STIM1 directly interacts with Orai1. The CAD domain of STIM1 (aa 342–448) is essential for the STIM1/Orai1 interaction and the activation of the CRAC channel which lead to Ca^2+^ influx and replenishment of the ER store.

In the present study, we show that STIM1 associates with *C*. *trachomatis* inclusion, where it co-localizes with CERT and VAPB at ER-Inclusion MCSs. Our data suggest that in infected cells, distinct pools of STIM1 engage in ER-PM MCSs or ER-Inclusion MCSs, but the minimal domain of STIM1 required for enrichment in both types of MCSs appears to be conserved. Altogether this study identified STIM1 as a novel component of ER-*C*. *trachomatis* inclusion MCSs and we discuss its role(s) in the developmental cycle of the bacteria.

## Materials and Methods

### Ethics statement

All genetic manipulations and containment work were approved by the Yale Biological Committed and are in compliance with the section III-D-1-a of the National Institutes of Health guidelines for research involving recombinant DNA molecules.

### Cell lines and bacterial strains

HeLa cells were obtained from ATCC (CCL-2) and cultured at 37°C with 5% CO_2_ in DMEM high glucose (Invitrogen) supplemented with 10% heat inactivated FBS (Invitrogen). *C*. *trachomatis Lymphogranuloma venereum*, *Type II* were obtained from ATCC (L2/434/Bu VR-902B) and used throughout the study, unless otherwise noted. The *C*. *trachomatis* CtL2 GFP(IncD) strain was described previously [24]. The *C*. *trachomatis* CtL2 CFP(Gro) strain expresses the cyan fluorescent protein (CFP) from the *groESL* operon promoter and terminator and the corresponding CFP reporter was designed and cloned as previously described for the *C*. *trachomatis* CtL2 mCherry(Gro) strain [12]. *Chlamydia caviae* was obtained from Roger Rank (University of Arkansas). *Chlamydia* propagation and infection were performed as previously described [25].

### Plasmid construction

Restriction enzymes and T4 DNA ligase were obtained from New England Biolabs (Ipswich, MA). PCR was performed using Herculase DNA polymerase (Stratagene). PCR primers were obtained from Integrated DNA Technologies and their sequence is listed in S1 Table. mCh-STIM1 (kind gift from R. Lewis, Stanford, CA) and all truncated and internal deletion were cloned into the *EcoR*I and *Xho*I restriction sites of pCDNA3.1+. R. Lewis also kindly provided the HRP-STIM1 construct. pGFP-Sec61ß and pGFP-Rtn3C were obtained from T. Rapoport (Harvard Medical School, MA) and Orai1-GFP was obtained from B. Baird (Cornell University, NY). YFP-CERT and CFP-VAPB were described previously [12].

### DNA transfection

DNA transfection was performed using Fugene 6 according to the manufacturer recommendations.

### Immunofluorescence and microscopy

At the indicated times, the cells seeded onto glass coverslips were fixed for 30 min in PBS containing 4% paraformaldehyde. Immuno-staining was performed at room temperature. Antibodies were diluted in PBS containing 0.1% BSA and 0.1% Triton X-100. Samples were washed with PBS and examined under an epifluorescence or spinning disc confocal microscope.

### Antibodies

The following primary antibodies were used: rabbit polyclonal anti-*C*. *trachomatis* IncA (1:200, kindly provided by T. Hackstadt, Rocky Mountain Laboratories), anti-*C*. *caviae* IncA (1:300, kindly provided by A. Subtil, Pasteur Institute, Paris, France) and mouse anti-STIM1 (1:100, BD Biosciences).

The following secondary antibodies were used: goat anti-rabbit AlexaFluor 514 antibody (1:1,000, Molecular Probes) and goat anti-mouse AlexaFluor 488 antibody (1:1,000, Molecular Probes).

### Electron Microscopy and peroxidase cytochemistry

HeLa cells transiently expressing HRP-STIM1 were infected for 24h and processed as follows. Samples were fixed in 1.2% glutaraldehyde in 0.1M sodium cacodylate buffer pH7.4 for 1 hour at room temperature. Samples were rinsed 3 times in sodium cacodylate buffer and incubated in 0.1M ammonium phosphate for 10min, which is replaced with filtered 0.1M ammonium phosphate containing 0.5mg/ml diaminobenzidine (DAB) + 0.005% hydrogen peroxide until a brown color develops in the sample. Samples were rinsed in cold water and post-fixed in 1% osmium tetroxide/1% potassium ferrocyanide for 1 hour. Samples were then rinsed and en bloc stained in aqueous 2% uranyl acetate for a further hour followed by rinsing, dehydrating in ethanol and infiltrated with Embed 812 (Electron Microscopy Sciences) resin and baked over night at 60°C. Hardened blocked were cut using a Leica UltraCut UC7. 60nm sections were collected and stained using 2% uranyl acetate and lead citrate. Samples were all viewed FEI Tencai Biotwin TEM at 80Kv. Images were taken using Morada CCD and iTEM (Olympus) software.

### Time-lapse video microscopy and Ca^2+^ store depletion

HeLa cells were seeded on 35-mm imaging dishes (MatTek, Ashland, MA) and transfected with the mCh-STIM1 construct 12hrs prior to infection with a *C*. *trachomatis* strain expressing GFP. 24h post infection, cells expressing low level of mCh-STIM1 were selected and imaged every 2.5 min before and after addition of 2 μM Thapsigargin (Tg), on a Nikon TE2000E spinning disc confocal microscope equipped with a humidified live cell environmental chamber set at 37°C and 5% CO_2_. The Volocity software (Improvision, Lexington, MA) was used to analyze and process the data.

### Quantification of the fraction of inclusion membrane covered by various mCh-STIM1 constructs

The quantification of the fraction of inclusion membrane covered by various mChSTIM1 constructs was done using the Volocity software on images acquired with a spinning disc confocal microscope. For each inclusion, the quantification was performed on a stack of XY planes corresponding to a 2.2 μm slice located in the middle of the inclusions of interest. The volume corresponding to the mCh-STIM1 signal associated with the inclusion membrane was measured and normalized to the length of inclusion membrane. Full length STIM1 was set to 100%.

For each mCh-STIM1 construct, data from three biological (10–20 inclusions each) and three experimental replicates were analyzed by using Prism (Version 5.0a, GraphPad software, Inc.). Statistical analysis was performed by using a Student’s t test.

### STIM1 depletion

The protocol for siRNA transfection has been described previously [25]. STIM1 depletion was performed by transfection of a pool of four siRNA duplexes that led to an 87 +- 2% reduction in the level of STIM1 mRNA level normalized to GAPDH expression levels. The sequences of the STIM1 siRNA duplexes were: CAUCAGAAGUAUACAAUUG, AGAAGGAGCUAGAAUCUCA, AGGUGGAGGUGCAAUAUUA, GGUGGUGUCUAUCGUUAUU.

### Inclusion size quantification and computer-assisted image analysis

siRNA treated cells were infected with *C*. *trachomatis* expressing mCherry under the *groESL* operon promoter [12] and fixed 36 hr p.i. The nuclei were labeled with the DNA dye Hoechst. The cells were subjected to automated fluorescence microscopy to capture images corresponding to the cell nuclei and the inclusion. Computer-assisted image analysis, using the analytical tools of the MetaMorph software, was used to determine the number of nuclei and the surface area of each inclusion.

### Infectious progeny production

HeLa cells incubated with the indicated siRNA duplexes for 3 days were collected 48h post infection with a strain of *C*. *trachomatis* expressing mCherry under the *groESL* operon promoter [12], lysed with water and dilutions of the lysate were used to infect fresh HeLa cells. The cells were fixed 24h post infection and the number of inclusion forming units (IFUs) was determined after assessment of the number of infected cells.

### Inhibition of *C*. *trachomatis* protein synthesis

At the indicated time post infection, *C*. *trachomatis* infected cells were incubated in the presence of 50μg/ml of Chloramphenicol. The chloramphenicol was kept for the duration of the experiment.

## Results

### STIM1 is enriched in patches at *C*. *trachomatis* inclusion membrane

**Fig 1:**
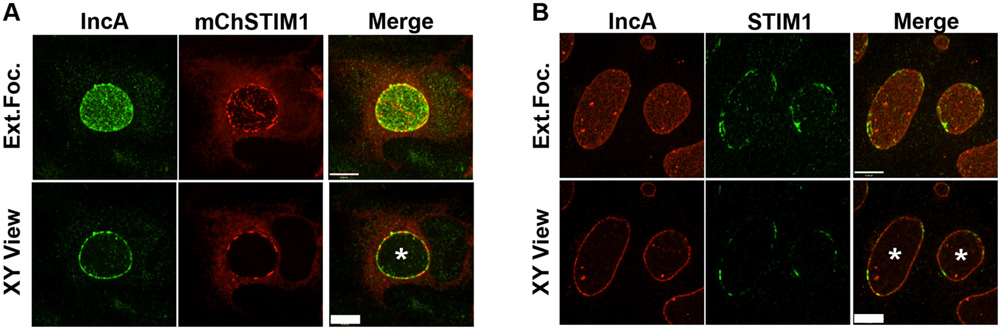
STIM1 is enriched in patches at the *C*. *trachomatis* inclusion membrane. Confocal micrographs of HeLa cells infected with *C*. *trachomatis* for 24h and stained using antibodies against the *C*. *trachomatis* inclusion membrane protein IncA (A-B) (left panels, IncA, green (A) and red (B)). The cells were transfected with a mCherrySTIM1 construct 18h pre-infection (A) (middle panels, mChSTIM1, red) or stained using antibodies against STIM1 (B) (middle panels, STIM1, green). The merge is shown on the right. The top and bottom panels respectively correspond to the extended focus view combining all the confocal planes (Ext.Foc.) and a single plane crossing the middle of the inclusion (XY View). The asterisk in the XY View Merge panel indicates the inclusion. Scale bar: 10μm.

We have previously shown that ER-Inclusion MCSs were formed during *C*. *trachomatis* infection and that CERT and VAPB, two components of ER-Golgi MCSs, localized to these platforms [5]. We investigated if any other known components of MCSs also associated with the inclusion and found that STIM1, a component of ER-PM MCSs, was enriched at the *C*. *trachomatis* inclusion membrane (Fig 1). When expressed in HeLa cells, mCherry-STIM1 localized to the bulk of the ER and co-localized with the general ER marker Sec61ß (S1 Fig). In *C*. *trachomatis* infected HeLa cells expressing a mCherry-STIM1 construct, STIM1 also localized to the bulk of the ER (Fig 1A, mChSTIM1, please note that the signal is not as strong as in S1 Fig because of lower level of expression). In addition, labeling of the inclusion membrane, using anti-IncA antibodies (Fig 1A, IncA), revealed a pool of STIM1 molecules that was highly enriched in small patches in the vicinity of the inclusion membrane. Small patches of endogenous STIM1 were also detected in close association with the inclusion membrane in *C*. *trachomatis* infected cells immuno-stained with antibodies against STIM1 and IncA (Fig 1B). Altogether, these results indicate that STIM1 is enriched at the inclusion membrane.

### The general ER markers Rtn3C and Sec61ß are not enriched in the vicinity of the *C*. *trachomatis* inclusion

**Fig 2:**
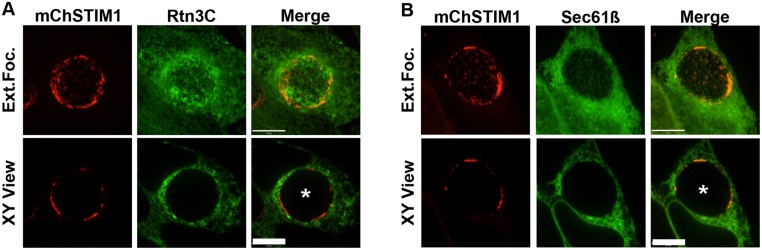
The ER markers Rtn3C and Sec61ß are not enriched at the *C*. *trachomatis* inclusion membrane. Confocal micrographs of HeLa cells co-expressing mCherrySTIM1 (A-B) (left panels, mChSTIM1, red) and GFP-Rtn3C (A) (middle panels, Rtn3C, green) or GFP-Sec61ß (B) (middle panels, Sec61ß, green) and infected with *C*. *trachomatis* for 24h. The merge is shown on the right. The top and bottom panels respectively correspond to the extended focus view combining all the confocal planes (Ext.Foc.) and a single plane crossing the middle of the inclusion (XY View). The asterisk in the XY View Merge panel indicates the inclusion. Scale bar: 10μm.

We next investigated whether ER proteins were generally enriched at the inclusion membrane by determining the cellular localization of two well established ER-resident proteins, the reticulon component Rtn3C and Sec61ß, in infected cells (Fig 2). In *C*. *trachomatis* infected HeLa cells co-expressing mChSTIM1 and GFP-Rtn3C (Fig 2A) or mChSTIM1 and GFP-Sec61ß (Fig 2B), STIM1 was enriched in patches at the inclusion as shown in Fig 1. Rtn3C and Sec61ß localized to the bulk of the ER and were found in proximity of the inclusion. However, in comparison to STIM1, Rtn3C and Sec61ß did not appear enriched in the vicinity of the inclusion. Altogether these results suggest that distinct pools of ER-resident proteins associate with the inclusion membrane. ER markers, such as Rtn3C or Sec61ß, appear to be distributed at the same level throughout the ER, while STIM1 is highly enriched in ER patches contacting the inclusion membrane.

### STIM1 is enriched at CERT- and VAPB-positive ER-Inclusion MCSs

**Fig 3:**
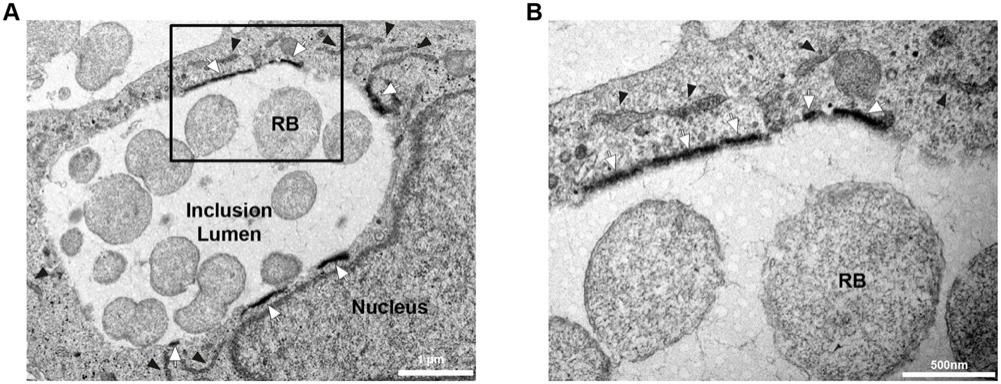
STIM1 is enriched at ER-Inclusion MCSs. Electron micrographs of HeLa cells expressing HRP-STIM1, infected with *C*. *trachomatis* for 24h and processed for conventional transmission electron microscopy coupled with peroxidase cytochemistry. White arrows and black arrowheads respectively indicate ER structures displaying high and low level of HRP-STIM1. The area outline by the box in A is shown at higher magnification in B. RB: Reticulate Body. Scale Bars: 1 μm (A), 500 nm (B).

The pattern of STIM1 association with *C*. *trachomatis* inclusion was reminiscent of the CERT and VAPB patterns observed at ER-Inclusion MCSs [5, 6]. This observation led us to investigate whether STIM1 also localized to ER-Inclusion MCSs. We used conventional transmission electron microscopy coupled with peroxidase cytochemistry of *C*. *trachomatis* infected HeLa cells expressing HRP-STIM1 (Horse Radish Peroxidase-STIM1). STIM1 is a multi-domain protein with a single trans-membrane domain. The N-terminus localizes within the ER lumen and the C-terminus faces the cytosol. The HRP-STIM1 construct restricts the peroxidase reaction to the lumen of the ER and HRP-STIM1-positive ER structures appear filled with a black signal [26, 27]. As shown in Fig 3, multiple STIM1-positive structures were detected in close apposition to the inclusion membrane (Fig 3, white arrows), confirming the localization of STIM1 to ER-Inclusion MCSs. Moreover, in agreement with STIM1 enrichment to these platforms, the peroxidase reaction was more pronounced at ER-Inclusion MCSs than in cytosolic ER structures that were further away from the inclusion membrane (Fig 3, black arrowheads).

**Fig 4:**
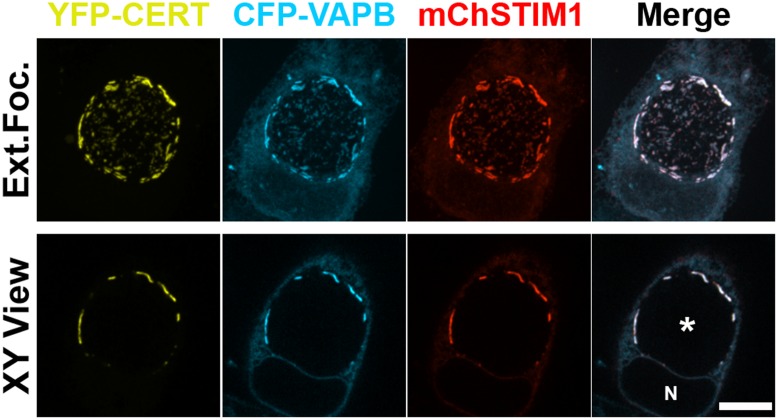
STIM1 colocalizes with CERT and VAPB at the *C*. *trachomatis* inclusion membrane. Confocal micrographs of HeLa cells co-expressing YFP-CERT (yellow), CFP-VAPB (cyan) and mCherrySTIM1 (red) and infected with *C*. *trachomatis* for 24h. The merge is shown on the right. The top and bottom panels respectively correspond to the extended focus view combining all the confocal planes (Ext.Foc.) and a single plane crossing the middle of the inclusion (XY View). The asterisk in the XY View Merge panel indicates the inclusion. N: Nucleus. Scale bar: 10μm.

We next investigated whether STIM1 co-localized with CERT/VAPB at ER-inclusion MCSs. HeLa cells co-expressing YFP-VAPB, CFP-CERT and mChSTIM1 were analyzed by confocal fluorescence microscopy 24h post infection with *C*. *trachomatis* (Fig 4). As observed previously [5, 6], CERT and VAPB were enriched in patches at *C*. *trachomatis* inclusion. Importantly, STIM1 co-localized with the VAPB- and CERT-positive patches, indicating that all three proteins were recruited to the same pool of ER-Inclusion MCSs. Altogether these results indicate that STIM1 is enriched at CERT- and VAPB-positive ER-Inclusion MCSs.

### STIM1 is associated with *C*. *trachomatis* inclusion throughout the developmental cycle

**Fig 5:**
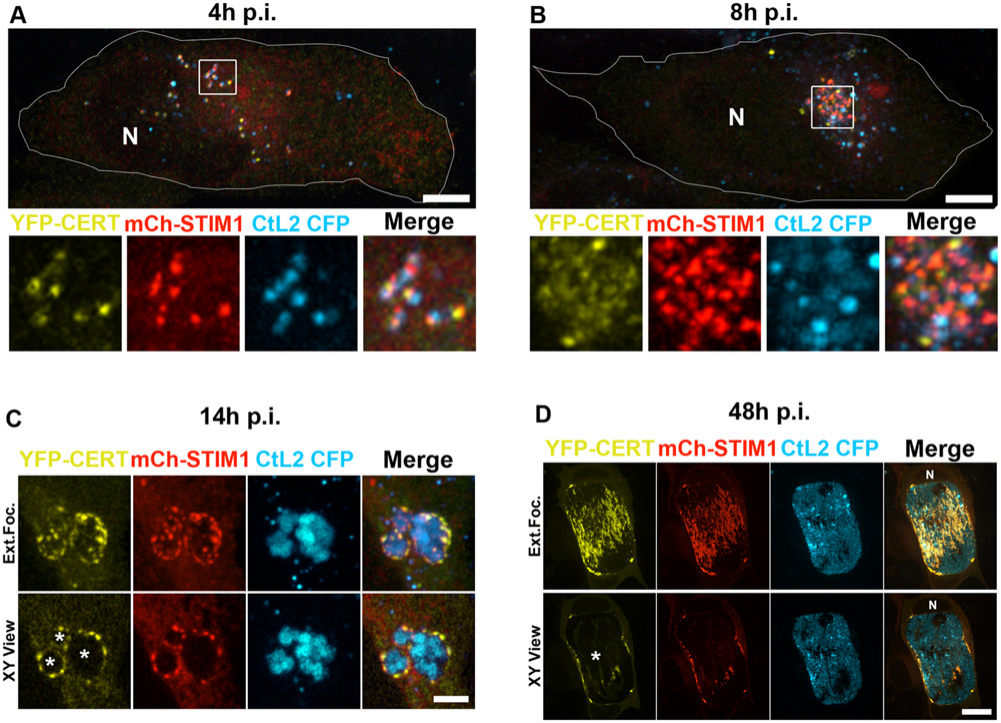
STIM1 is associated with the *C*. *trachomatis* inclusion membrane throughout the developmental cycle. A-B. Confocal micrographs of HeLa cells co-expressing YFP-CERT (yellow) and mCherrySTIM1 (mCh-STIM1, red) and infected with *C*. *trachomatis* expressing CFP (CtL2 CFP, cyan) for 4h (A), 8h (B). The extended focus view of 10 0.2 μm individual planes crossing the middle of the cell is shown. The boundary of the cell and the nucleus (N) are outlined and the area outlined by a box is shown at higher magnification below the whole cell micrograph. The merge is shown on the right. Scale bar: 10 μm. C-D. HeLa cells, as described in A and B, were infected for 14h (C) or 48h (D). Three small inclusions that have not yet fused are shown (C). The merge is shown on the right. The top and bottom panels respectively correspond to the extended focus view combining all the confocal planes (Ext.Foc.) and a single plane crossing the middle of the inclusion (XY View). The asterisk in the XY View Merge panel indicates the inclusions. N: Nucleus. Scale bars: 4μm (C), 20μm (D).

CERT association with the inclusion was detected as early as 2 hours post infection and the interaction remained throughout the developmental cycle [5, 6]. We therefore investigated whether STIM1 association with the inclusion followed the same dynamics. HeLa cells co-expressing YFP-CERT and mChSTIM1 were infected with a strain of *C*. *trachomatis* expressing CFP and the association of CERT and STIM1 with the inclusion was analyzed by confocal fluorescence microcopy 4 and 8 hours post infection (Fig 5). At 4h post infection, EBs appeared as small and bright CFP-positive dots scattered throughout the cytosol (Fig 5A). Because of the low level of expression, CERT and STIM1 were barely visible in the cytosol of the host cell. Nevertheless, both proteins appeared as bright puncta closely apposed to the EBs. CERT and STIM1 co-localization was imperfect at that time, but both markers clearly localized to the same side of the EBs, most likely where the ER is starting to contact the inclusion membrane and initiate the formation of ER-Inclusion MCSs. By 8h post infection, the EBs had clustered to the perinuclear area of the cell and started to differentiate back into RBs, as indicated by their increase in size and their more diffuse cyan signal, most likely due to dilution of the cyan protein that was pre-packed into the EBs (Fig 5B). As observed 4h post infection, CERT and STIM1 were mostly detected in puncta in close association with the bacteria. The association of the endogenous STIM1 with *C*. *trachomatis* inclusion was also detected 7h and 10h p.i. (S2 Fig). By 14h post infection, the cells harbored multiple inclusions that displayed CERT and STIM1 positive-patches on their surface (Fig 5C). STIM1 and CERT co-localization was more pronounced at that time and resembled the pattern observed 24h post infection (Fig 4), suggesting that the formation of ER-Inclusion MCSs was complete. The inclusions were still positive for CERT and STIM1 30h post infection (not shown). By 48h post infection, the cells harbored very large inclusions that occupied most of the cytosol and still displayed large patches positive for STIM1 and CERT (Fig 5D). Despite the late time point, the inclusion membrane was not directly apposed to the plasma membrane (Fig 5C XY view) indicating that STIM1 and CERT were still specifically associated with the inclusion rather than being pushed onto the inclusion membrane because of space constraint. Altogether these results indicate that CERT and STIM1 associate concomitantly with the nascent inclusion and remain associated throughout *C*. *trachomatis* developmental cycle.

### STIM1 cellular localization in response to Ca^2+^ store depletion

**Fig 6:**
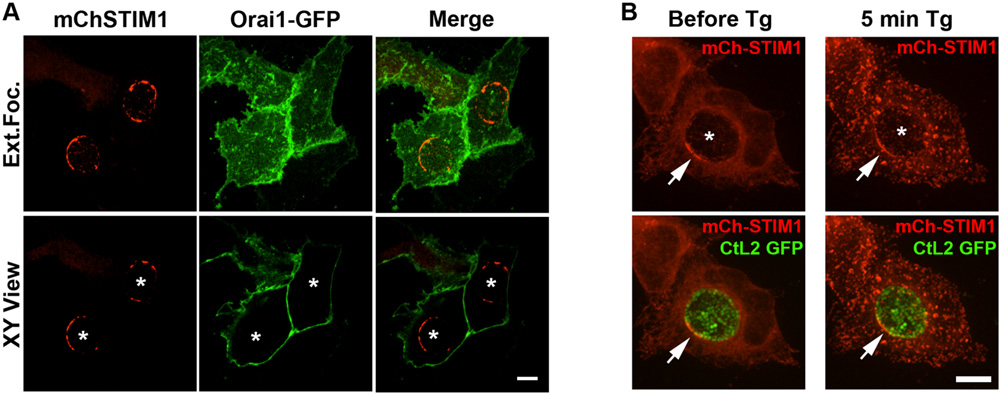
STIM1 cellular localization in response to Ca^2+^ store depletion. A. Confocal micrographs of HeLa cells co-expressing mCherry-STIM1 (left panels, mChSTIM1, red) and Orai1-GFP (middle panels, Orai1-GFP, green) and infected with *C*. *trachomatis* for 24h. The merge is shown on the right. The top and bottom panels respectively correspond to the extended focus view combining all the confocal planes (Ext.Foc.) and a single plane crossing the middle of the inclusion (XY View). The asterisks in the XY View panels indicate the position of the inclusions. Scale bar: 10μm. B. Confocal micrographs of live HeLa cells expressing mCherry-STIM1 (mCh-STIM1, red) and infected for 24h with a strain of *C*. *trachomatis* that expresses GFP (CtL2 GFP, green). The images were acquired before (left panels, Before Tg) and 5 min after addition of Thapsigargin (Tg) (right panels, 5min Tg). The top and bottom panels respectively correspond to the mCh-STIM1 signal alone and the merge. An extended focus view combining all the confocal planes is shown. The arrow indicates STIM1 enrichment at ER-Inclusion MCSs. The asterisk indicates the position of the inclusion. Scale bar: 10μm.

As shown previously, upon Ca^2+^ store depletion, STIM1 relocalized to ER-PM MCSs, where it co-localized with the PM Ca^2+^ channel composed of oligomerized Orai1 molecules ([28] and S3 Fig). To determine whether the STIM1-Orai1 interaction also occurred at ER-Inclusion MCSs, HeLa cells co-expressing mChSTIM1 and GFP-Orai1 were infected with *C*. *trachomatis* for 24h and analyzed by confocal fluorescence microscopy (Fig 6A). As expected, STIM1 was enriched in patches surrounding the inclusion. Orai1 however localized exclusively to the PM and did not co-localize with the STIM1-positive patches on the inclusion. These results indicate that STIM1 enrichment at ER-Inclusion MCSs is independent of Orai1 and suggest that different pools of STIM1 molecules localize to ER-Inclusion MCSs or ER-PM MCSs. To confirm this hypothesis, HeLa cells expressing mChSTIM1 and infected with *C*. *trachomatis* for 24h were subjected to Ca^2+^ store depletion by addition of Thapsigargin (Tg). The cellular localization of STIM1 was monitored by time-lapse video microscopy (Fig 6B). As expected, in resting cells, STIM1 localized to the bulk of the ER and was enriched in patches at the inclusion membrane. Upon Tg addition, the STIM1 molecules quickly concentrated to patches, similar to the STIM1/Orai1 patches observed in uninfected cells treated with Tg (S3 Fig). STIM1 was still detected at the inclusion membrane, suggesting that the pool of STIM1 molecules engaged in ER-Inclusion MCSs prior to Tg addition remained associated with the inclusion. Altogether, these results indicate that compared to the pool of STIM1 molecules that localizes to ER-PM MCSs, the pool engaged in ER-Inclusion MCSs does not interact with Orai1 and remains associated with the inclusion, even upon Ca^2+^ store depletion. In addition, STIM1 subversion during *C*. *trachomatis* infection does not impair the host cell response to Ca^2+^ store depletion.

### The CAD domain of STIM1 is required for STIM1 localization to ER-Inclusion MCSs

**Fig 7:**
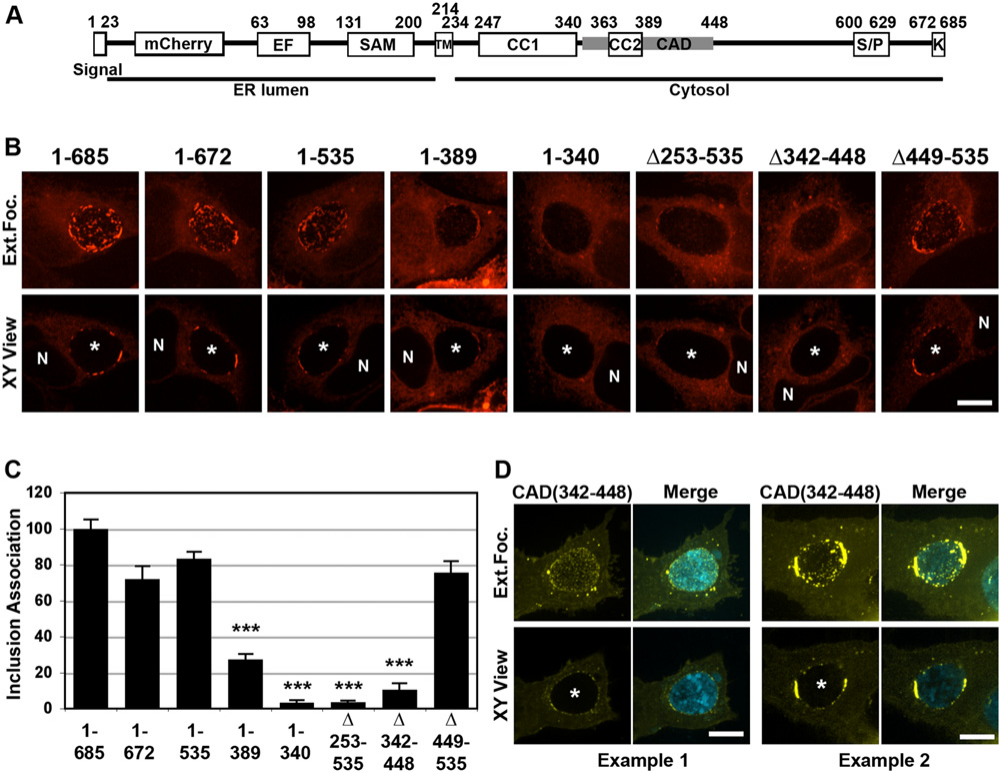
The CAD domain of STIM1 is required for STIM1 localization to ER-Inclusion MCSs. A. Schematic representation of the major domains of the STIM1 protein, their respective amino acid residue position and their respective cellular localization (ER lumen or Cytosol). Signal: signal peptide; EF: EF-hand; SAM: sterile alpha motif; TM: transmembrane domain; CC1: coiled-coil 1; CC2: coiled-coil 2; CAD: CRAC activation domain; S/P: Serine-proline-rich region; K: lysine-rich region. B. Confocal micrographs of HeLa cells expressing the indicated mCherry-STIM1 construct (red) and infected with *C*. *trachomatis* for 24h. The top and bottom panels respectively correspond to the extended focus view combining all the confocal planes (Ext.Foc.) and a single plane crossing the middle of the inclusion (XY View). The asterisk in the XY View Merge panel indicates the inclusion and N indicates the nucleus. Scale bar: 10μm. C. Quantification of inclusion association of the indicated mCh-STIM1 constructs compared to full-length mCh-STIM1. *** p value <0.001. D. Confocal micrographs of HeLa cells expressing YFP-CAD (CAD(342–448), yellow), and infected for 24h with a strain of *C*. *trachomatis* expressing CFP (cyan). The merge is shown on the right. Two representative examples of YFP-CAD pattern on the inclusion are shown. The top and bottom panels respectively correspond to the extended focus view combining all the confocal planes (Ext.Foc.) and a single plane crossing the middle of the inclusion (XY View). The asterisk in the XY View Merge panel indicates the inclusion. Scale bar: 10μm.

We next sought to identify the minimal domain of STIM1 required for localization to ER-Inclusion MCSs. STIM1 is a multi-domain protein with the N-terminus residing in the ER lumen and the C-terminus facing the cytosol (Fig 7A). A series of C-terminal and internal truncated variants were generated within STIM1 cytosolic domain. The ability of each STIM1 construct to cluster in patches in the vicinity of the inclusion was assayed by confocal microscopy (Fig 7B) and the inclusion association of each truncated variant was quantified (see Materials and Methods) and compared to the full-length STIM1 construct (Fig 7C). Deletion of Lys-rich region (STIM1 1–672) and the Ser/Pro-rich region (STIM1 1–535) did not affect STIM1 localization to the inclusion. Deletion up to the second coiled-coil (CC2) (STIM1 1–389) reduced STIM1 association with the inclusion by 70% and further deletion of the first coiled-coil (CC1) (STIM1 1–340) abolished STIM1 enrichment to the inclusion. A similar result was observed with a construct harboring an internal deletion spanning amino acid 253 and 535 (STIM1 Δ253–535). Shorter internal deletion within this domain indicated that deletion of the CAD domain (STIM1 Δ342–448), but not the downstream domain (STIM1 Δ449–535), abolished STIM1 enrichment at the inclusion. Altogether, these results indicate that the CAD domain of STIM1 (342–448) is required for STIM1 enrichment to the inclusion. To determine whether the CAD domain was sufficient for STIM1 localization to the inclusion, we assayed the cellular localization of YFP-CAD in cells infected with *C*. *trachomatis* (Fig 7D). When expressed at high level, YFP-CAD formed aggregates (not shown). However, in low level expressing cells, YFP-CAD was detected in association with the inclusion in a pattern resembling the one of full length STIM1. In some instances, the CAD signal appeared evenly distributed in small patches on the inclusion (Fig 7D, example 1), while some inclusions displayed larger patches highly enriched for the CAD domain (Fig 7D, example 2). Altogether, these results indicate that the CAD domain is necessary and probably sufficient for STIM1 association with *C*. *trachomatis* inclusion.

### STIM1 depletion does not affect *C*. *trachomatis* growth

**Fig 8:**
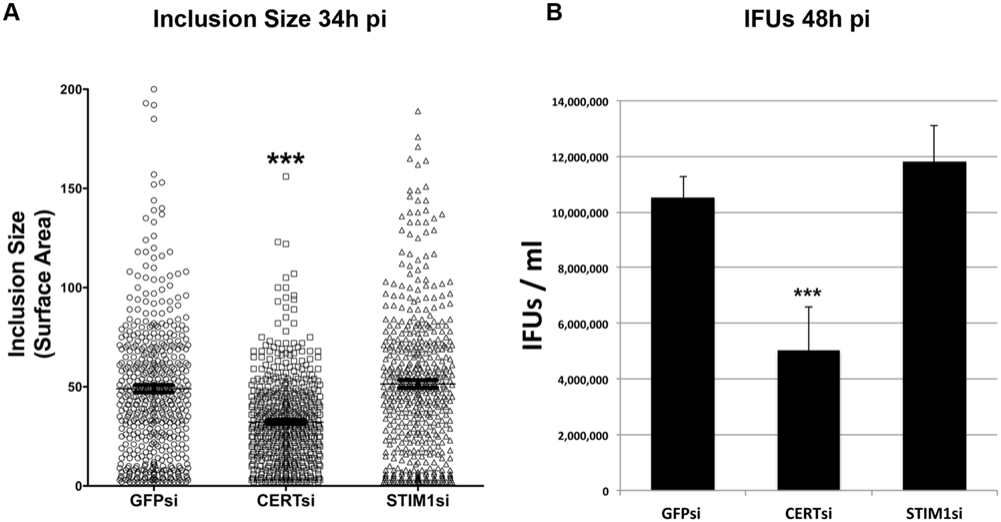
STIM1 depletion does not affect *C*. *trachomatis* growth. HeLa cells were transfected with control siRNA (GFPsi) or a pool of CERT siRNA (CERTsi) or a pool of siRNA against STIM1 (STIM1si) for 3 days and infected with a strain of *C*. *trachomatis* expressing mCherry. (A) For each condition, the surface area of the inclusions was determined (Arbitrary Units). Each circle, square or triangle represents data from a single inclusion. (B) The number of infectious bacteria (IFUs/ml) was determined 48h p.i. (A-B) Data show the mean and standard deviation of biological triplicate of a representative experiment. ***P<0.0001 (student’s t test).

Finally, we investigated if STIM1 depletion had any impact on *C*. *trachomatis* intracellular growth. STIM1 was depleted using a pool of 4 siRNA duplexes and *C*. *trachomatis* replication, based on the size of the inclusions and the production of infectious progeny, was compared to cells transfected with control siRNA duplexes targetting GFP or CERT expression (Fig 8). As observed previously, compared to GFP-depleted control cells, CERT depletion led to a decrease in the size of the inclusions 24h p.i. (Fig 8A, CERTsi) and in the number of infectious bacteria recovered 48 p.i. (Fig 8B, CERTsi) [5]. STIM1 depletion however did not affect *C*. *trachomatis* replication (Fig 8A and 8B, STIM1si).

## Discussion

During *Chlamydia* infection, the ER is found in the vicinity of the pathogen-containing inclusions and *bona fide* MCSs between the ER and the inclusion membrane are formed [4–7]. While we agree with Dumoux *et*. *al*. that general ER markers are present in closed vicinity of the inclusion, our data suggest that only a subset of ER-resident proteins, such as VAPA/B, are specifically enriched at the inclusion compared to the bulk of the ER. Here, we introduce a novel ER-resident protein specifically enriched at ER-Inclusion MCSs, STIM1.

### Composition of ER-*Chlamydia trachomatis* Inclusion MCSs

In cells, the protein composition of MCSs is specific to the identity of the contacting organelles. For example, the ER integral protein VAPA and VAPB and the soluble ceramide transfer protein CERT localize to ER-Golgi MCSs [29], while ER-PM MCSs are enriched in Orai, a PM protein forming a Ca^2+^ channel, and the ER Ca^2+^ sensor STIM1 [11]. Components of MCSs therefore determine their function and ER-Golgi MCSs and ER-PM MCSs have respectively been implicated in the non-vesicular trafficking of lipids from the ER to the Golgi and Ca^2+^ homeostasis.

We had shown previously that the ER-Inclusion MCSs formed during *C*. *trachomatis* infection harbor the host ceramide transfer protein CERT and the ER integral membrane proteins VAPA and VAPB [5, 12]. ER-Inclusion MCSs therefore resemble ER-Golgi MCSs, except that the inclusion membrane substitutes for the Golgi membrane. We and the Engel lab therefore proposed that the ER-Inclusion MCSs may constitute an alternative pathway for lipid acquisition by *C*. *trachomatis*, *via* sphingomyelin synthesis directly at the inclusion membrane [5, 6].

**Fig 9:**
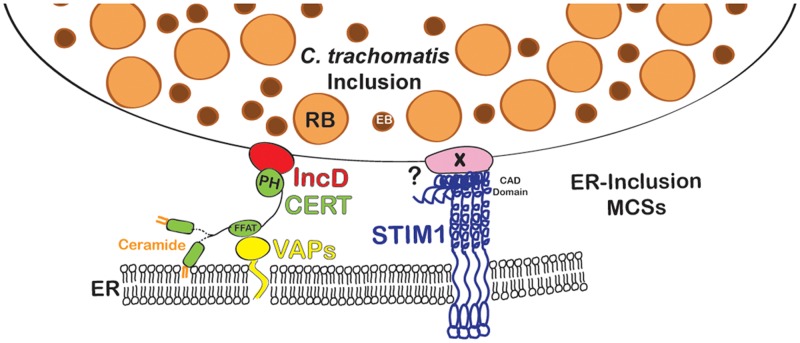
Schematic representation of ER-Inclusion MCSs. CERT (green) localizes to ER-Inclusion MCSs. The PH domain of CERT binds to the *C*. *trachomatis* inclusion membrane protein IncD (red) and the FFAT motif of CERT allows CERT association with VAPA/B (Yellow) on the ER. In addition to CERT, VAPA/B and IncD, the ER Calcium sensor STIM1 (dark blue) also localizes to ER-Inclusion MCSs. The CAD domain of STIM1 is required for association with the inclusion. Future work will determine whether a *Chlamydia* factor X (light pink) is involved in this process.

In the present study, we showed that STIM1, a component of ER-PM MCSs, localized to ER-Inclusion MCSs. This observation thus opened the possibility that, in addition to the assembly of MCSs functionally equivalent to ER-Golgi MCSs, the inclusion membrane may support the assembly of MCSs sharing similarities with ER-PM MCSs. However, we found that Orai1, the STIM1 interacting partner at ER-PM MCSs, did not localize to the inclusion membrane. Moreover, CERT and STIM1 co-localized at the inclusion membrane as early as 4h post infection and remained associated throughout the development cycle. These results thus argue against the existence of two classes of ER-Inclusion MCSs, harboring either CERT/VAP or STIM1 and instead suggest that the ER-inclusion MCSs formed during *C*. *trachomatis* infection are hybrid MCSs composed of proteins usually found at ER-Golgi or ER-PM MCSs (Fig 9).

### *Chlamydia* effector proteins and ER-Inclusion MCSs

Our data illustrate the ability of *C*. *trachomatis* to efficiently manipulate its environment to redirect cellular factors of unrelated functions (e.g. CERT and STIM1, as reported here) to the same structures. The insertion of the *C*. *trachomatis* effector protein IncD into the inclusion membrane supports CERT recruitment to the inclusion [5], and by analogy, we suggest the existence of a *C*. *trachomatis* effector protein involved in STIM1 recruitment. Interestingly, we found that the CAD domain of STIM1, which faces the cytosol and is required for STIM1 interaction with Orai1, is also important for STIM1 association with *C*. *trachomatis* inclusion membrane. One attractive hypothesis is that *C*. *trachomatis* encodes an inclusion membrane effector that mimic Orai1 and allows for STIM1 recruitment to the inclusion (Fig 9).

The notion that *C*. *trachomatis* specific effector(s) is/are involved in STIM1 recruitment to the inclusion membrane is reinforced by the fact that STIM1 did not associate with *C*. *caviae* inclusion (S4A Fig). This species specificity for host factors recruitment to the inclusion has been reported previously for the Rab proteins [30], and also for CERT ([5] and S4B Fig). *C*. *caviae* lacks *incD*, which provides an explanation for the absence of CERT recruitment to the inclusion. Moreover, we have shown here that *C*. *caviae* inclusions were also negative for STIM1. Altogether, these results indicate species specificity with regard to STIM1 recruitment to *Chlamydia* inclusion and provide further evidence that IncD/CERT/STIM1 are linked to similar structures. They also further highlight that *Chlamydia* species with different host and tissue tropism have evolved differently to manipulate their cellular environment and satisfy the needs imposed by their intracellular life style.

Finally, we attempted to inhibit bacterial protein synthesis during infection to address the role of potential *C*. *trachomatis* effector proteins in STIM1 recruitment to the inclusion. Because STIM1 associates with the inclusion early (4hp.i.) and throughout the developmental cycle, protein synthesis was either inhibited at the early- (1h-10hp.i.) or mid-stages (16hp.i.). It was previously reported that inhibition of *Chlamydia* protein synthesis during the early stages of the developmental cycle prevented the migration of the inclusions to the MTOC (microtubule-organizing center) due to an effect on inclusion membrane modification [31]. When protein synthesis was inhibited 1h or 2h post infection, the inclusions failed to cluster at the MTOC by 10h p.i. and were STIM1-negative at 4h, 6h, 8h and 10h p.i. (not shown). This result suggested that *de novo* protein synthesis is required for STIM1 recruitment to *C*. *trachomatis* inclusion, either through a general effect on inclusion membrane maturation and/or the direct effect of a specific *C*. *trachomatis* factor. When protein synthesis was inhibited after STIM1 association with the inclusion (4h, 6h or 8hp.i.), the nascent inclusions clustered at the MTOC and remained STIM1-positive up to 10h p.i. (latest time-point examined), as shown in Fig 5 when bacterial protein synthesis was not inhibited. Moreover, when protein synthesis was inhibited 16h p.i., after maturation of the inclusion and recruitment of STIM1, the inclusion did not increase in size and STIM1 remained associated with the inclusion up to 48h p.i. (S5 Fig, top panels). These results suggest that, once recruited to *C*. *trachomatis* inclusion, STIM1 remains associated with the inclusion membrane even in the absence of *Chlamydia* protein synthesis. We note that the IncD-dependent association of CERT with the inclusion [5] was also maintained after *C*. *trachomatis* protein synthesis inhibition (S5 Fig, bottom panels). Thus, additional work will be required to determine whether putative *C*. *trachomatis* effector(s) are required for the recruitment of STIM1 to the inclusion membrane.

### STIM1 and host-pathogen interaction

STIM1 has been linked to *Clostridium difficile* and rotaviruses pathogenesis [32, 33]. Upon contact of *C*. *difficile* with the host cell, the actin-ADP-ribosylating toxin *C*. *difficile* transferase (CDT) induces an Orai/STIM1-dependent Ca^2+^ influx resulting in the enrichment of the PM in adhesion molecules leading to an increase in *C*. *difficile* adhesion and invasion. In the case of rotaviruses, the virus-encoded NSP4 protein localizes to the ER where it function as a viroporin (i.e. a virus-encoded pore-forming or ion channel protein) and induces ER Ca^2+^ store depletion leading to the virus-induced constitutive activation of STIM1/Orai1 and the influx of Ca^2+^ necessary for virus replication.

*C*. *difficile* and rotaviruses therefore take advantage of the ability of STIM1 to activate Orai1 in order to increase cytosolic Ca^2+^ concentration and activate Ca^2+^-dependent signaling events that are beneficial for host subversion. Our data indicate that *C*. *trachomatis* infection does not induce STIM1 relocalization to ER-PM MCSs and that the pool of STIM1 engaged at ER-Inclusion MCSs is not associated with Orai1. It is therefore unlikely that, as described for *C*. *difficile* or the rotaviruses, STIM1 plays a role in Ca^2+^ influx into the cytosol. Ca^2+^ however does play a role during *C*. *trachomatis* infection. For example, the calcium-dependent cytosolic phospholipase A2 (cPLA2) has been implicated in lipid acquisition, bacterial growth and induction of a cell autonomous cellular immunity [34, 35]. It was also proposed that Ca^2+^ channels (IP3-R and SERCA2) and Ca^2+^ accumulate at the inclusion [36]. It is therefore possible that *C*. *trachomatis* infection triggers the local release of Ca^2+^ from the ER in the vicinity of the inclusion. This could have two consequences: 1) the local activation of Ca^2+^-dependent signaling pathways and 2) the local release of Ca^2+^ from STIM1 EF-hand and the clustering of STIM1 molecules at ER-Inclusion MCSs. If this model would explain the mechanisms of STIM1 enrichment at ER-Inclusion MCSs, the function of STIM1 during *C*. *trachomatis* infection remains to be determined. Independently of its Ca^2+^ sensing properties STIM1 could, in concert with other bacterial and/or host proteins, act as a tether/stabilizer of ER-Inclusion MCSs. Our attempts to detect a defect in bacterial replication or ER-Inclusion MCSs formation in STIM1-depleted cells have so far been unsuccessful and may suggest potential redundancy.

### *Chlamydia* subversion of the host cell through the formation of MCSs

Similar to *C*. *trachomatis*, the inclusions formed by the *Chlamydia*-related organisms, *Simkania negevensis* [37, 38] and *Waddlia chondrophila* [39, 40] are also closely associated with the ER. Unlike the situation observed for *C*. *trachomatis*, however, the interaction of *Simkania* and *Waddlia* inclusions with the ER correlates with an association with mitochondria. *Simkania* inclusion membrane is in close contact with both organelles, whereas *Waddlia* inclusions associate with mitochondria that in turn interact with the ER. Altogether, these studies suggest that ER-Inclusion (*C*. *trachomatis*, *Simkania*), and potentially Mitochondria-Inclusion (*Simkania*, *Waddlia*) and ER-Mitochondria (*Waddlia*, potentially *Simkania*) MCSs may be formed during infection with *Chlamydia*-related organisms. These results reinforce the idea that MCSs between pathogen-containing vacuoles and organelles are important for the intracellular survival and development of pathogens. Besides nutrient and lipid acquisition, these points of contact have been proposed to inhibit the ER stress response during *Simkania* infection [37]. In addition, MCSs may participate to the innate immune response mechanisms and mediate the STING-dependent sensing of pathogens-derived compound [41]. As the list of host and bacterial factors that localize to these platforms expend, we will get a better understanding of their function(s) and gain more insight on the molecular mechanisms that support *Chlamydia* interaction with the host cell.

## Supporting Information

S1 Fig mCh-STIM1 localizes to the bulk of the ER and co-localizes with Sec61ß. Confocal micrographs of HeLa cells co-expressing mCh-STIM1 (top panels, red) and Sec61ß-GFP (middle panels. green). The merge is shown in the bottom panels. A whole cell is shown in the left panels and the right panels show a higher magnification of the outlined area. Scale Bar: 20μm (whole cell), 5μm (Inset).
(TIF)

S2 Fig Endogenous STIM1 associates with the inclusion in the early stages of the developmental cycle. Confocal micrographs of HeLa cells infected with a strain of *C*. *trachomatis* expressing CFP (Blue) for 7h (top panels) and 10h (bottom panels) and stained with anti-STIM1 antibodies (red). The merge is shown on the right. Scale Bar 20μm.
(TIF)

S3 Fig Calcium store depletion induces the formation of STIM1- and Orai1-containing ER-PM MCSs. Confocal micrographs of HeLa cells expressing mChSTIM1 (red) alone (left panels) or mChSTIM1 and Orai1-GFP (green) (right panels) at resting state (A) or 8min after Ca2+ store depletion by addition of Thapsigargin (B). The top and bottom panels respectively correspond to the extended focus view combining all the confocal planes (Ext.Foc.) and a single plane corresponding to the Plasma Membrane (XY PM). The merge is shown on the right. Scale Bar: 20μm.
(TIF)

S4 Fig STIM1 is not recruited to *C*. *caviae* inclusion. Confocal micrographs of HeLa cells expressing mCherry-STIM1 (red) (A) or YFP-CERT (yellow) (B), infected with *C*. *caviae* for 24h and stained with anti-IncA antibodies (yellow) (A), (red) (B). The merge is shown on the right. Scale Bar 10μm.
(TIF)

S5 Fig STIM1 and CERT remain associated with *C*. *trachomatis* inclusion after inhibition of bacterial protein synthesis. Confocal micrographs of HeLa cells expressing mCherry STIM1 (top panels) or YPF-CERT (bottom panels) and infected with *C*. *trachomatis* for 48h. The infected cells were incubated (right panels) or not (left panels) with 50μg/ml Chloramphenicol starting 16h p.i. to block bacterial protein synthesis. Scale Bar: 10μm.
(TIF)

S1 Table Primers used in this study. (PDF)
