# Supplementary figures and images for: STIM1 Is a Novel Component of ER-Chlamydia trachomatis Inclusion Membrane Contact Sites
Source: PLoS One. 2015 Apr 27;10(4):e0125671. doi: 10.1371/journal.pone.0125671 (PMC4411163; doi:10.1371/journal.pone.0125671)

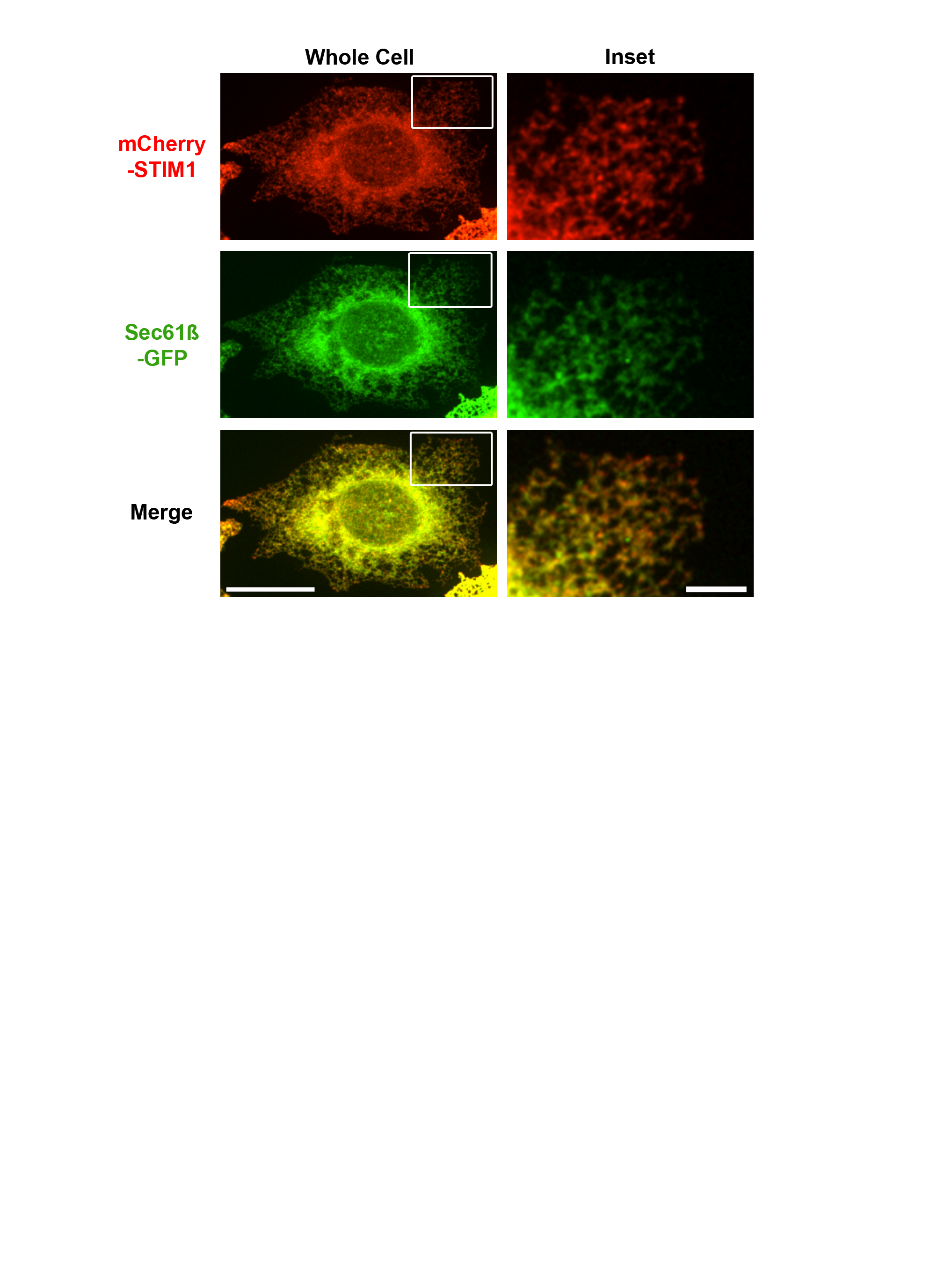

Supplement: S1 Fig — Confocal micrographs of HeLa cells co-expressing mCh-STIM1 (top panels, red) and Sec61ß-GFP (middle panels. green). The merge is shown in the bottom panels. A whole cell is shown in the left panels and the right panels show a higher magnification of the outlined area. Scale Bar: 20μm (whole cell), 5μm (Inset). (TIF) [file pone.0125671.s001.tif]

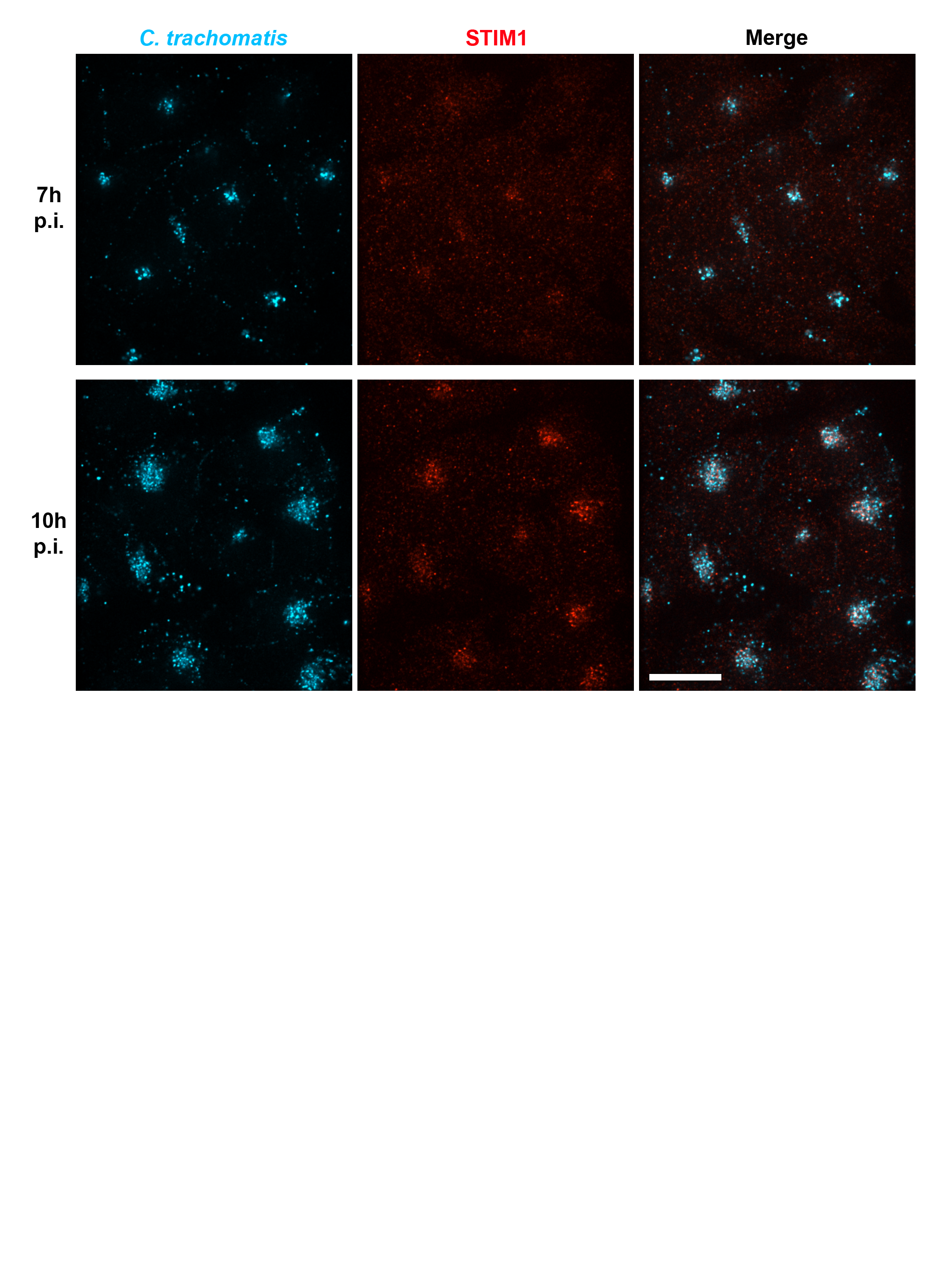

Supplement: S2 Fig — Confocal micrographs of HeLa cells infected with a strain of C. trachomatis expressing CFP (Blue) for 7h (top panels) and 10h (bottom panels) and stained with anti-STIM1 antibodies (red). The merge is shown on the right. Scale Bar 20μm. (TIF) [file pone.0125671.s002.tif]

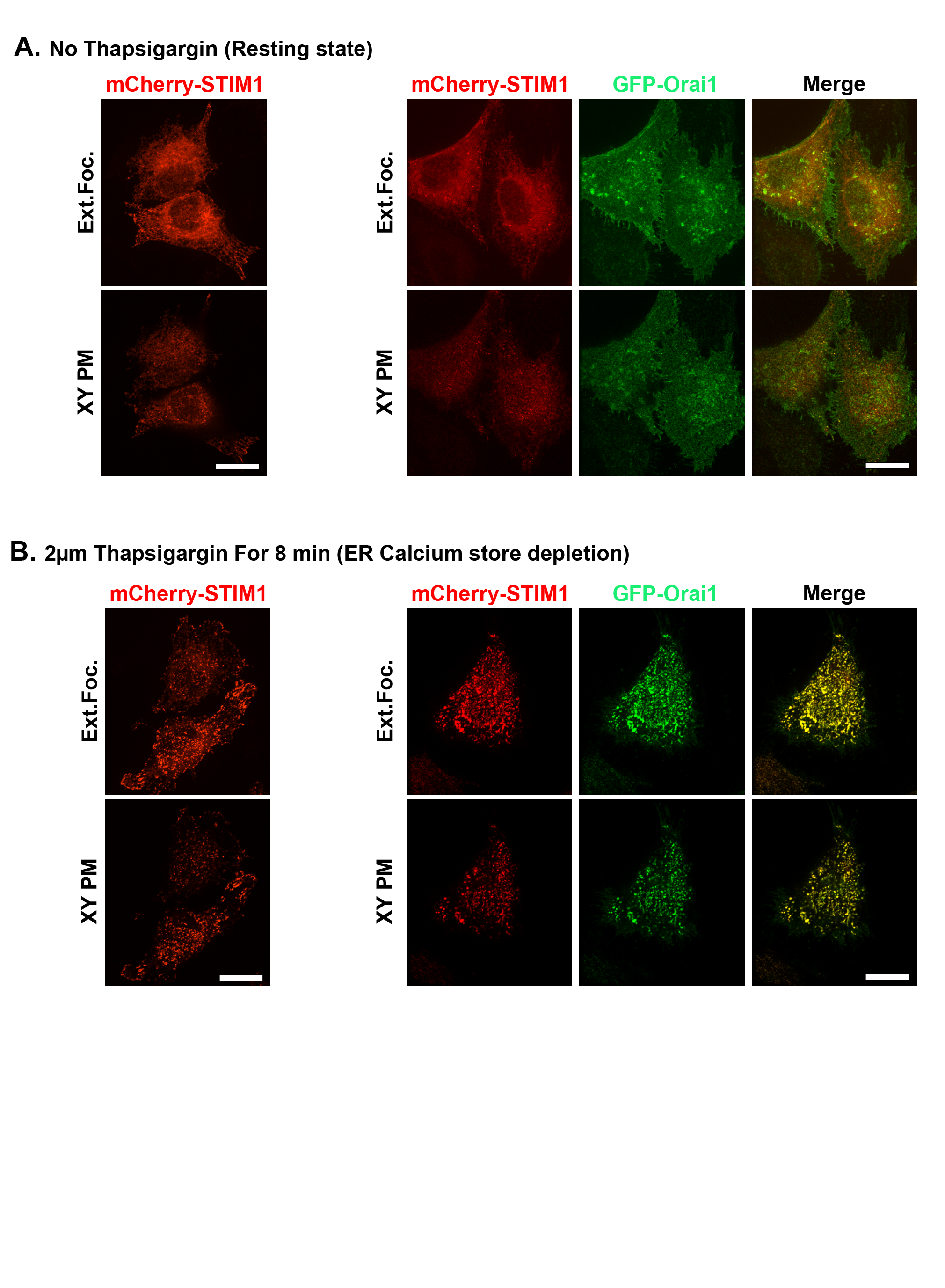

Supplement: S3 Fig — Confocal micrographs of HeLa cells expressing mChSTIM1 (red) alone (left panels) or mChSTIM1 and Orai1-GFP (green) (right panels) at resting state (A) or 8min after Ca2+ store depletion by addition of Thapsigargin (B). The top and bottom panels respectively correspond to the extended focus view combining all the confocal planes (Ext.Foc.) and a single plane corresponding to the Plasma Membrane (XY PM). The merge is shown on the right. Scale Bar: 20μm. (TIF) [file pone.0125671.s003.tif]

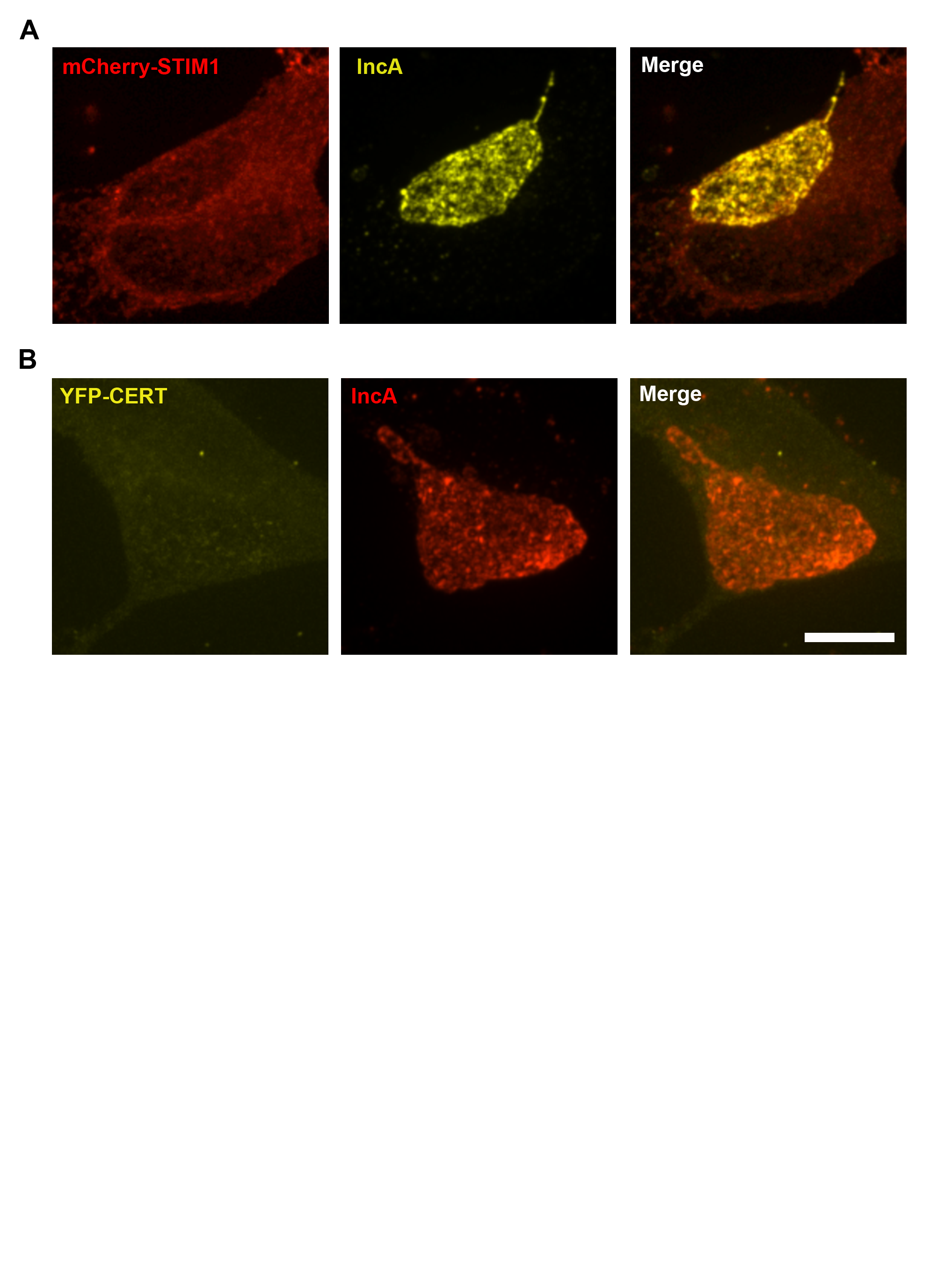

Supplement: S4 Fig — Confocal micrographs of HeLa cells expressing mCherry-STIM1 (red) (A) or YFP-CERT (yellow) (B), infected with C. caviae for 24h and stained with anti-IncA antibodies (yellow) (A), (red) (B). The merge is shown on the right. Scale Bar 10μm. (TIF) [file pone.0125671.s004.tif]

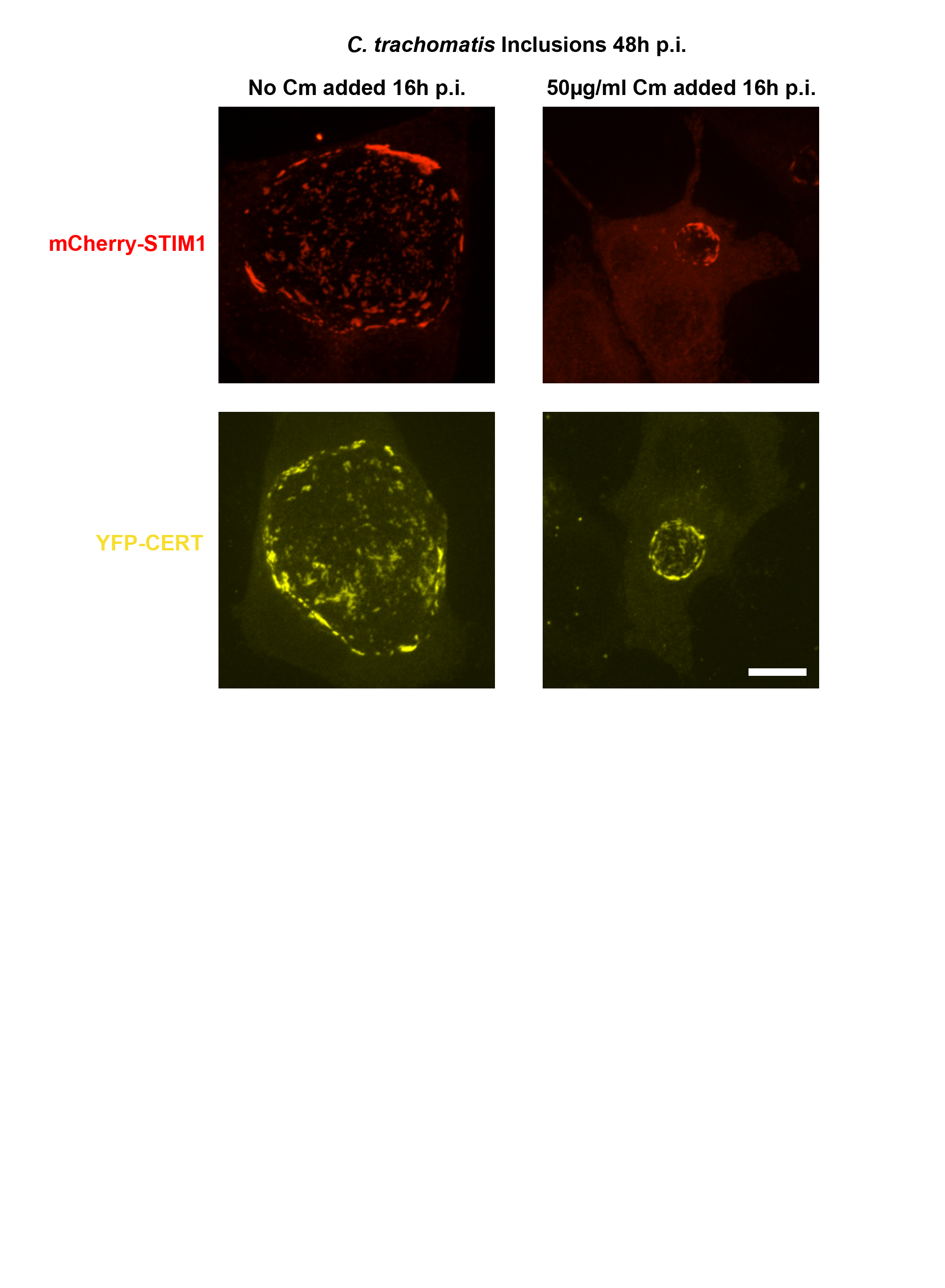

Supplement: S5 Fig — Confocal micrographs of HeLa cells expressing mCherry STIM1 (top panels) or YPF-CERT (bottom panels) and infected with C. trachomatis for 48h. The infected cells were incubated (right panels) or not (left panels) with 50μg/ml Chloramphenicol starting 16h p.i. to block bacterial protein synthesis. Scale Bar: 10μm. (TIF) [file pone.0125671.s005.tif]
