# Supplementary material for: STIM1 Is a Novel Component of ER-Chlamydia trachomatis Inclusion Membrane Contact Sites
Source: PLoS One. 2015 Apr 27;10(4):e0125671. doi: 10.1371/journal.pone.0125671 (PMC4411163; doi:10.1371/journal.pone.0125671)
Supplement: S1 Table — (PDF) [file pone.0125671.s006.pdf]

S1 Table: Primers used in this study.

**PRIMER NAME**	**PRIMER SEQUENCE**
STIM1 5Eco	GAAGAATTCatggatgtatgcgtccgtcttgccc
STIM1 685 3Xho	CTCCTCGAGctacttcttaagaggcttcttaaag
STIM1 672 3Xho	CTCCTCGAGCTAccggcctgggctggagtctg
STIM1 535 3Xho	CTCCTCGAGCTAgggggccacacgctggcggtcac
STIM1 389 3Xho	CTCCTCGAGCTAtgtgtttctcttctttttatcttc
STIM1 340 3Xho	CTCCTCGAGCTAtgagctgtgagattctagctcc
STIM1 Delta 253-535 5	gaaggacttggaggggttacacaaacctcctcagatgagccgtgc
STIM1 Delta 253-535 3	gcacggctcatctgaggaggtttgtgtaacccctccaagtccttc
STIM1 Delta 342-448 5	agctagaatctcacagctcatggtcactggtggctgccctcaacatag
STIM1 Delta 342-448 3	ctatgttgagggcagccaccagtgaccatgagctgtgagattctagc
STIM1 Delta 449-535 5	gtcaacaaccctggcatccacaaacctcctcagatgagccgtgc
STIM1 Delta 449-535 3	gcacggctcatctgaggaggtttgtggatgccagggttgttgac
